# “Sewing Is Part of Our Tradition”: A Case Study of Sewing as a Strategy for Arts-Based Inquiry in Health Research With Inuit Women

**DOI:** 10.1177/10497323211042869

**Published:** 2021-10-04

**Authors:** Laura Jane Brubacher, Cate E. Dewey, Naomi Tatty, Gwen K. Healey Akearok, Ashlee Cunsolo, Sally Humphries, Sherilee L. Harper

**Affiliations:** 1 University of Guelph, Guelph, Ontario, Canada; 2 Iqaluit, Nunavut, Canada; 3 Qaujigiartiit Health Research Centre, Iqaluit, Nunavut, Canada; 4 Labrador Institute of Memorial University, Happy Valley-Goose Bay, Labrador, Canada; 5 University of Alberta, Edmonton, Alberta, Canada

**Keywords:** arts-based research, qualitative health research, Inuit, Nunavut, sewing

## Abstract

In this article, we present a case study of sewing as a strategy for arts-based inquiry in health research, situated within a broader project that highlighted Nunavut Inuit women’s childbirth experiences. Five focus groups were hosted as sewing sessions with pregnant women (*N* = 19) in Iqaluit, Nunavut (2017–2018). Women’s reflections on the sessions, and the significance of sewing to Inuit, were integrated with researchers’ critical reflections to examine the value of sewing as a strategy for arts-based inquiry within a focus group method: results related to the flexibility of the sessions; how collective sewing created space for voicing, sharing, and relating; sewing as a tactile and place-specific practice tied to Inuit knowledge and tradition; and lessons learned. Our results underscore the possibilities of arts-based approaches, such as sewing, to enhance data gathering within a focus group method and to contribute to more locally appropriate, place-based methods for Indigenous health research.

## Introduction

*Sewing is part of our tradition, our culture. It gives a warm welcoming into the group. It really puts your mind focused on our group. It’s grounding. Once you start sewing, you forget about everything around you. Like being out on the land. You get away from technology, you get away from everything. Like out on the land—you get away from reality, and you receive what the land has to offer you, what the animals in the water are. You see a seal, and it’s swimming very calmly, smoothly in the water when you see it. It’s nothing chaotic out there. It’s very peaceful. You don’t hear the city. You don’t hear anything. All you hear is the motor of the boat. Or the sound of the skidoo. Or the snow. It’s just a real peaceful state, your entire body and soul is feeling around you. That’s how it feels also with sewing. ’Cause you’re keeping the tradition going, you have all these different reasons to be motivated to be able to participate in these sewing groups. Not only that, you feel proud in the end of your accomplishment. It’s such a satisfying feeling of emotion that, “Oh, I finished this! It’s going to be useful. It’s going to be used and kept warm.” Something you can provide for your family*. (Naomi Tatty, Inuk team member and co-author, on sewing)

Arts-based approaches are increasingly emergent in health research and practice as creative, engaging, and empowering means to explore participants’ lived experiences of health issues, perspectives on health systems, and concepts of health (Archibald & Blines, 2021; Smit et al., 2021). Researchers have used drawing (Caldairou-Bessette et al., 2020), photography (Watchman et al., 2020; Woodgate et al., 2021), visual artifacts (Talsi et al., 2021), and filmmaking (Baumann et al., 2020), for instance, as modes for both generating health research data and mobilizing results. These arts-based approaches are increasingly recognized as both valuable approaches to inquiry in their own right (Boydell et al., 2012) and also as strategies to use concurrently with or embedded within more conventional qualitative methods, such as interviews or focus group discussions (Fraser & al Sayah, 2011). Indeed, the interface of the arts and health research is expansive, evolving, and an imaginative space within which researchers may continue to discover new methodological possibilities that broaden our understandings of health and well-being (Boydell et al., 2012). For populations with rich artistic traditions—such as many Indigenous communities worldwide—arts-based approaches often align with important and culturally embedded forms of knowledge-sharing (Flicker et al., 2014), and thus have potential to facilitate rich dialogue within the data gathering process (Boydell et al., 2012). For instance, researchers have used photovoice (Harper et al., 2015; Lardeau et al., 2011), collaborative podcasting (Day et al., 2017), digital storytelling (Cunsolo Willox et al., 2013; Harper et al., 2012), and participatory film (Borish et al., 2021; MacDonald et al., 2015) to engage Indigenous Peoples in community-based and community-led, action-oriented health research (Bell et al., 2021; Hammond et al., 2018).

Importantly, when working with Indigenous communities and with Indigenous Peoples, Kovach (2009) discusses the process of gathering data through “making and doing” as resonating with Indigenous epistemologies, ontologies, and axiologies. Indeed, to avoid a continuation of the “colonial project” in research (Smith, 2012), researchers face an ethical imperative to adapt their methods to reflect Indigenous ways of knowing and being (Simonds & Christopher, 2013); privilege the voices, perspectives, and worldview of Indigenous participants (Chilisa, 2011); and engage in research rooted in “relational accountability,” reciprocity, and respect (Wilson, 2008, p. 77). While in some arts-based approaches the art form also constitutes the data, such as digital media in photovoice (Castleden et al., 2008) or digital storytelling (Cunsolo Willox et al., 2013; Harper et al., 2012), this research utilized sewing as a strategy for arts-based inquiry to facilitate knowledge-sharing and data gathering, not as a data source or knowledge mobilization product. This aligns with Boydell et al.’s (2012) definition of arts-based research as the “use of any art form (or combinations thereof) at any point in the research process in generating, interpreting, and/or communicating knowledge” (p. 3) as well as Leavy’s (2018) use of the term “arts-based research” as “an umbrella category that encompasses all artistic approaches to research” (p. 4). While sewing has previously been used in arts-based research with Indigenous Peoples (Healey, 2019; Jackson & Coleman, 2015), opportunity exists for further exploring this strategy for arts-based inquiry to facilitate data gathering within an Inuit context.

We explore the methodological possibilities and value of arts-based inquiry in health research through a case study on maternal health in Iqaluit, Nunavut. Specifically, this article (a) critically analyzes the process of using sewing as a strategy for arts-based inquiry to enhance focus groups with pregnant Inuit women in Iqaluit, (b) explores sewing as a strategy for arts-based inquiry that is embedded in Nunavut Inuit culture and context, and (c) evaluates the use of sewing as a strategy for arts-based inquiry in an Indigenous health context and identifies key “lessons learned” that have relevance to researchers who may employ this strategy. In so doing, we draw connections from our research process to the broader context of critical public health research and praxis, particularly with Indigenous populations.

## Method

### Placing the Work: Birthing in Nunavut

**Figure 1.**
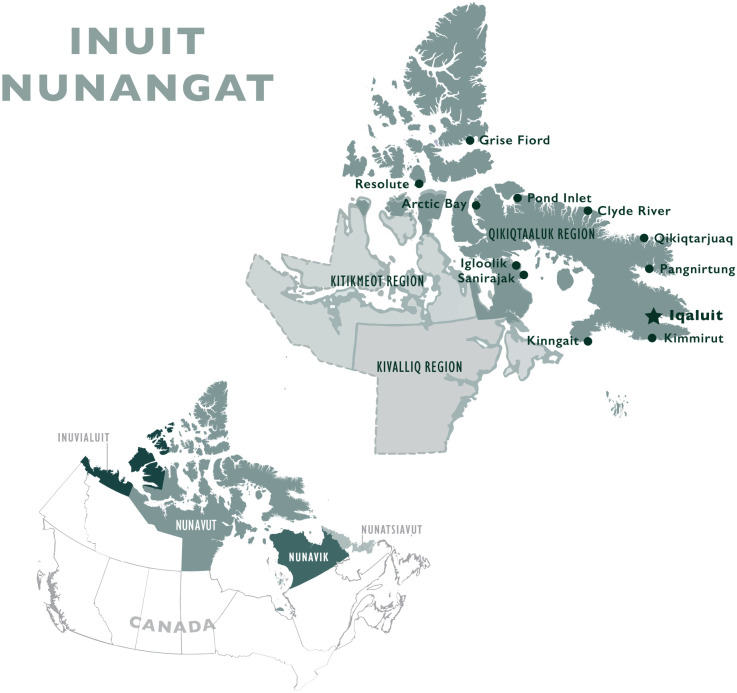
Map of Inuit Nunangat (Inuit Homelands), including the Inuit territory of Nunavut and the territorial capital city of Iqaluit, where this research was located.

This research focused on childbirth in the Inuit territory of Nunavut, within Inuit Nunangat (Inuit Homelands; Figure 1). Nunavut is a vast area, inclusive of 2.093 million km^2^ of lands and waters that Inuit have lived on and with for thousands of years (Mancini Billson & Mancini, 2007). Within Nunavut are 25 fly-in communities, located in three regions (from West to East): Kitikmeot, Kivalliq, and Qikiqtaaluk. Eighty-four percent (83.8%) of Nunavut’s population, of approximately 36,000, identify as Inuit (Statistics Canada, 2017).

**Figure 2.**
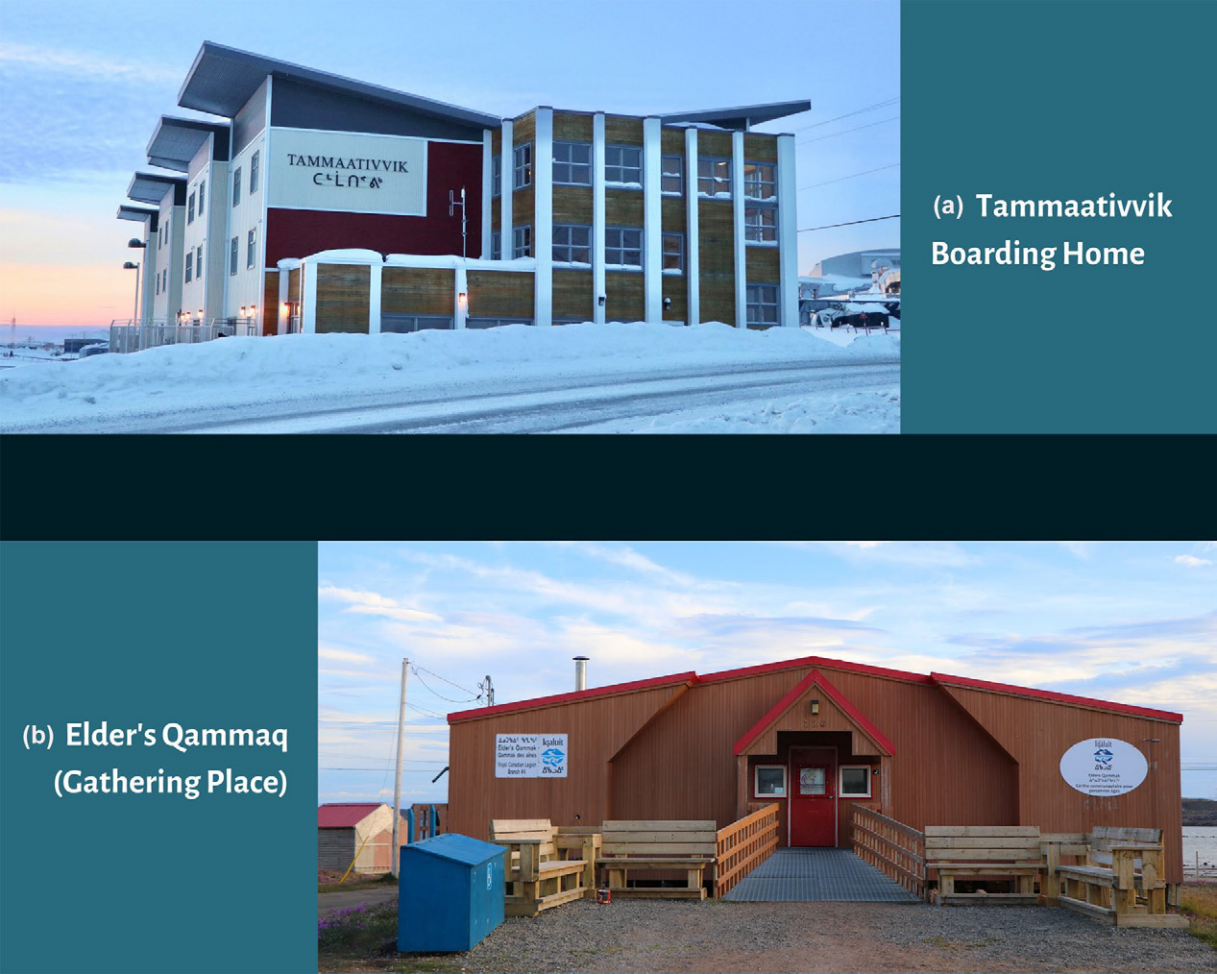
(a) Tammaativvik Boarding Home and (b) Elder’s *Qammaq* in Iqaluit, Nunavut, Canada, locations where sewing sessions were held during data collection between 2017 and 2018.

This research was located in the territorial capital of Iqaluit, a hub for maternity travel (“obstetric evacuation”)^1^ in Eastern Nunavut. Women within Qikiqtaaluk Region communities fly to Iqaluit for delivery at approximately 36 weeks’ gestation (Figure 1). While awaiting delivery, women stay at one of three residences in Iqaluit, one of which is the Tammaativvik Boarding Home (Figure 2), unless they require care at a tertiary care center in Ottawa. This model differs from the tradition of community-based birth and midwifery practiced by Inuit for millennia in the pre-colonial era (Kaufert & O’Neil, 1990). Over the last 50 years, birthing has transitioned from being located on the land to medical facilities to improve maternal and infant health (Healey & Meadows, 2007)—a medicalization of birthing that accompanied broader changes to health, economic, and administrative systems driven by colonial processes (Douglas, 2006). Inuit have long advocated for a return to community-based birthing and investment in midwifery training and regional birthing centers (McNiven, 2008; Pauktuutit Inuit Women’s Association of Canada, 1995).

Inuit have fulsome knowledge of birth-in-place; just as Inuit know and are of the land, so too are Inuit birthing practices intimately tied to place (Voisey et al., 1990).As the research team discussed possible modes for facilitating rich focus group conversations about pregnancy and childbirth, attention turned to another significant element to Inuit culture and livelihoods—sewing—which deeply connects Inuit to culture, spirituality, and kinship, familial, and ancestral relations (Aariak, 2018). Sewing also facilitates intergenerational teaching, learning, and dialogue between and among families and communities (Emanuelsen et al., 2020; Wachowich, 2018). Given this context, the hope was for sewing to be a research strategy that would resonate with pregnant Inuit women and create a more comfortable space for sharing. This exploration of sewing as a strategy for arts-based inquiry was situated within a multiyear, collaborative research project on the topic of Inuit women’s past birthing experiences in Nunavut and present experiences of obstetric evacuation. The larger project prioritized Inuit perspectives on birth and sought to also highlight Inuit perspectives on how birthing supports could be enhanced.

### Research Approach

This research project was guided by principles of community-based research (Israel et al., 2005; LaVeaux & Christopher, 2009), namely, that the research was designed, implemented, and analyzed by a team that included a Settler PhD candidate and an Inuk public health expert and researcher from Iqaluit, who provided methodological direction, facilitation of data gathering, and interpretation of results within the cultural, epistemological, and ontological context of the research, as well as a team of Northern and Southern-based academic researchers. This research was licensed by the Nunavut Research Institute (Licenses #01 024 17N-M; 01 012 18R-M; 01 016 19R-M; and 01 005 20R-M) and approved by the University of Guelph Research Ethics Board (Certificates #16NV049 and 16-12-718).

### Research Process

The research process consisted of four broad steps, including recruitment (gathering the group), data collection (gathering the data with a focus group method), language interpretation and data transcription, and data analysis. Sewing was a strategy for arts-based inquiry that facilitated and enhanced the data collection component of the research.

#### Gathering the group

The sewing sessions were facilitated by a female Inuk team member from Iqaluit and a female Settler PhD candidate. Two other female Inuit research associates and one Settler research associate were each involved in one session and helped with logistical preparations, including recruitment for one session hosted with women living in Iqaluit. For recruitment for the other four sessions, pregnant women currently residing at the Tammaativvik Boarding Home were personally invited and informed of the time and location of the sessions, and given the option of whether or not to attend. A range of two to seven pregnant women voluntarily attended and actively participated in each session (a total of 19 participants across five sessions). The composition of each group was fluid: Not all women stayed for the full 3-hour duration of each afternoon of sewing, as they sometimes had appointments to attend (Table 1). Participating women were from four communities in the Qikiqtaaluk Region of Nunavut.

**Table 1.** Details regarding the date of each two-part sewing session, the total number of participants per two-part session, as well as the composition of each group of women (e.g., the duration of participants’ attendance and other research associates present).

Date of Session	Total Number of Participants	Details on Composition of Group^a^
Session 1: August 2–3, 2017	3	• In addition to three participants: one Inuk research associate, one Settler research associate present
Session 2: August 5, 2017^b^	2	• In addition to two participants: two Inuit research associates present
Session 3: November 29–30, 2017	4	• Three participants attended only the first afternoon; one new participant attended the second afternoon
Session 4: February 28–March 1, 2018	7	• Seven participants attended the first afternoon; only three returned for the second afternoon
Session 5: April 17–18, 2018	3	• One participant left for an appointment partway through the first afternoon, then returned for the full duration of the second afternoon
Total	19	

a For all five sewing sessions, the same Inuk team member and Settler team member were present as facilitators (in addition to the “Total Number of Participants” and the additional individuals noted in this column). ^b^This session was hosted in a single afternoon (at the Elder’s *Qammaq*) with pregnant women living in Iqaluit.

#### Gathering the data

Data gathering occurred intermittently between 2017 and 2018, to include multiple, different groups of women. During this time, a total of four focus groups were held in the form of sewing sessions in a common room at the Tammaativvik Boarding Home (Figure 2a). Each session spanned two afternoons (approximately 6 hours total per session). In addition, one session was held in a single afternoon at the Elder’s *Qammaq* (Gathering Place) with pregnant women who regularly lived in Iqaluit (Figure 2b).

**Figure 3.**
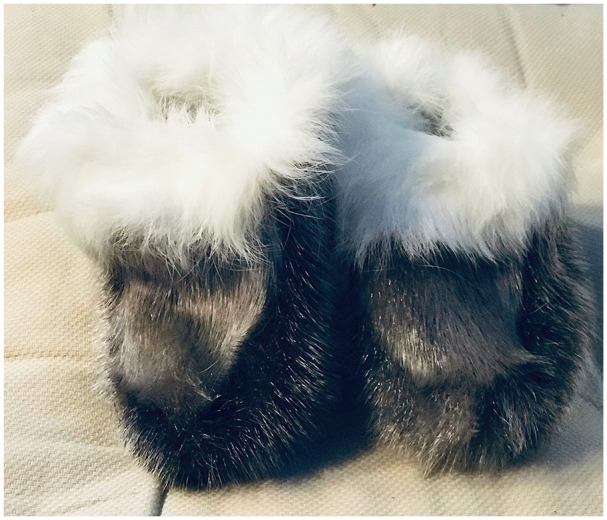
Photograph of sealskin baby slippers sewn in sewing sessions with pregnant women in 2017–2018. *Source.* Photograph courtesy of author.

Each session unfolded as a blend of sewing, conversation, storytelling, and moments of shared and companionable silence. All sewing materials were provided by the research team. Women sat in a circle and sewed sealskin baby slippers and mittens (Figure 3). An Inuk team member, who is a very knowledgeable seamstress, shared patterns with the women and assisted with their sewing, as needed. A Settler team member would open with a welcome and an introduction to the team and overarching project. Subsequently, participants provided verbal informed consent to participate in the research and, with permission, three audio recorders were turned on and situated around the circle. Then, a team member would pose open-ended, conversational questions to build familiarity within the group: for example, what communities women were from, when they arrived at the boarding home, how many children they have, and how they learned to sew. From there, while sewing, conversation was mostly unstructured. The research team would occasionally ask a question pertaining to four broad birthing-related research topics.

Data pertaining to participants’ views of the sewing sessions emerged organically as women commented on the sewing sessions as they unfolded or responded to a question from the team members; therefore, the 19 sewing session participants provided feedback on the sewing session approach. Three of these participants also responded to an invitation for a brief, individual, conversational follow-up interview within a day or two of the experience to further evaluate the sewing session. Concepts that informed the second section of results, related to “lessons learned” in this research, were drawn from the literature.

#### Language interpretation and data transcription

Sewing sessions were conducted in both English and Inuktitut. The language spoken emerged organically as women initiated or responded to conversation in one language or another. The gathered data consisted of audio recordings, which were transcribed verbatim by the research team (total recording time = 14.1 hours). The Inuktitut portions of the recordings were interpreted into English and subsequently transcribed and integrated among the English portions. To account for the challenge of interpreting Inuktitut concepts, and the potential for meaning to be lost, this process also involved a thorough debrief between team members. An Inuk team member interpreted to provide additional clarity and context to the recorded audio (Temple et al., 2006). This approach avoided “fixed—one word—translations” and, instead, produced “fluid descriptions of meanings” which helped contribute to the validity of this cross-language qualitative research (van Nes et al., 2010, p. 315).

#### Data analysis

**Figure 4.**
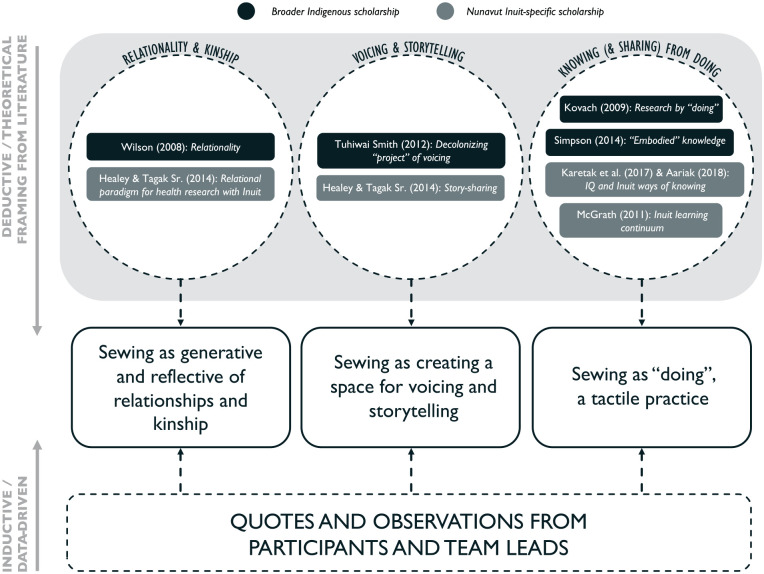
Visual representation of the analytical approach employed in this study’s qualitative analysis. *Note.* Themes were iteratively generated and refined using a hybrid inductive–deductive approach (Fereday & Muir-Cochrane, 2006), whereby the use of sewing as a strategy for arts-based inquiry was examined through the lens of the data and three categories of literature (in no particular order): (a) relationality and kinship, (b) voicing and storytelling, and (c) knowing (and sharing) from “doing.” Specific concepts from both the broader Indigenous scholarship and Inuit-specific scholarship that informed the generation and refinement of themes are denoted in the circles within each category.

Analysis of these qualitative data was a multilayered process that consisted of weaving together data from sewing sessions and interview transcripts, as well as annotations, memos, and written reflections from the team members, who critically reflected on and debriefed the sewing strategy in relation to the data gathered and the nuances of nonverbal cues, silence, facial expressions, and vocal tone and inflection in the audio recordings (Creswell & Miller, 2000). Initial open coding and annotating of the transcripts was done by hand (DeCuir-Gunby et al., 2011). Detailed memos and journaling were critical to synthesizing and making analytical links from the text to broader concepts (Birks et al., 2008). Further analysis was conducted in NVivo 12©, Version 12.1.0, and consisted of a thematic analysis, using a constant comparative method between and within transcripts (Braun & Clarke, 2006), and a hybrid inductive–deductive approach to iteratively generate and refine themes (Fereday & Muir-Cochrane, 2006) (Figure 4).

#### Theoretical framings

The use of sewing as a strategy for arts-based inquiry in an Indigenous health context more broadly (Obj.3)—and as a strategy for inquiry embedded in Nunavut Inuit culture in particular (Obj.2)—was examined through the lens of literature (both Inuit-specific scholarship and broader Indigenous scholarship) and the data. This literature can relate to three general categories, which provided theoretical framing to, and helped to generate and refine, themes from the data. Several results map directly onto these categories:

- *Relationality and kinship:* Wilson’s (2008) discussion of relationality, and the significance of relationships to Indigenous Peoples; Healey and Tagak’s (2014) discussion of a relational paradigm for health research with Inuit.
- *Voicing and storytelling:* Smith’s (2012) decolonizing “project” of voicing; importance of story-sharing within an Inuit context (Healey & Tagak, 2014).
- *Knowing (and sharing) from “doing”:* Creating research by “doing” (Kovach, 2009); an Inuit learning continuum (McGrath, 2011); Simpson’s discussion of embodied knowledge (Simpson, 2014); *Inuit Qaujimajatuqangit* (IQ) and Inuit ways of knowing (Aariak, 2018; Karetak et al., 2017).

## Results: Sewing as Research Strategy

The results presented relate to the significance of collective sewing for participants, and how sewing together facilitated data gathering. These broader themes provided the context for subsequent results on the particularities of how the sessions functioned and “lessons learned” from the process.

### Why and How Did Sewing Enhance Focus Groups?

Sewing as a strategy for arts-based inquiry was flexible and tactile, invited voicing and storytelling among participants, and reflected relationality and kinship. Sewing was described as place- and people-specific, and as an experience of embodying Inuit knowledge and tradition.

#### Sewing as a flexible and tactile practice

Our results indicated that sewing, as a tactile act of creation and imagination, enabled women to share in a different way. Less bounded by the parameters of a structured question–response format, the research team observed that sewing created flexibility for engaging in dialogue and storytelling as it naturally unfolded amid sewing. The concreteness of tying a knot and threading a needle were evident focal points for participants and team members alike and facilitated breaks in conversation. From the silence of sewing together, verbal sharing—when it occurred—was observed to be rich. The tactile experience of sewing was also described as a reminder of home for many women, a concrete practice that rooted women in a different place, emotionally. As one interviewee explained, “when you’re keeping busy, you forget about [family members] for a while, and enjoy yourself. You get less worries from them, doing something.”

#### Voicing and sharing

Women expressed their present and past experiences of pregnancy and childbirth to those gathered. They embodied a receptivity to each other’s stories, and, in turn, gave voice to their own experiences in the context of the group. For example, voicing concern about loved ones at home, one participant received validation and empathy from another, who responded with: “This will be over and done with eventually, and we’ll get home to them.” Both the particularities and shared commonalities of women’s lived realities of being away from home for childbirth were given voice; incarnate in word, tone, and inflection—and audible to those gathered—these stories were real, lived-in, and heard.

Excited and often humorous sharing of previous pregnancy and birthing experiences animated conversation around the circle, accompanied by ready sharing, hearty laughter, and tears. Many anecdotes shared in each session brought vibrancy and vitality to the dialogue. Stories from the past segued to stories of present pregnancies, and sharing of joys, as well as complications, from prior pregnancies. One woman who previously experienced a stillbirth reflected on her story of loss, and also of her memories of pregnancy and feelings therein. Women also shared their hopes and dreams for their unborn children, and vocally expressed what they imagined their children might be like. They audibly marveled at the strength of their bodies: “God blessed us with this amazing gift. I mean, we could grow human beings in our body, which men can’t do. I was like, ‘men are strong,’ and then I’m like, ‘no, women are strong!’” Women also told stories of the re-orienting connections they felt to their children:

**Participant 1:** Fall in love with the heartbeat, fall in love with the movement, fall in love with the ultrasound, fall in love with everything!
**Participant 2:** Changes your whole world. You see the whole world differently.

While women gathering to share experiences during pregnancy is not unique to this research, the inclusion of sewing as a strategy for arts-based inquiry that created a space that was generative, and an opportunity for the collective voicing and sharing of experiences while doing a familiar activity, enriched the focus group format, and allowed for participants to share organically and in a different manner than other types of focus groups would normally allow.

#### Relationality and kinship

The sewing sessions created new relationships both between and among participants and also between and among participants and local team members, who offered to take women berry picking and accompanied several women on a post-sewing group trip to a fabric store in town. Preexisting relationships were identified between participants, as women often discovered common friends or kinship relations. “That’s my [insert family member]’s [relation]” was a commonly echoed refrain around the room. While interpreting some Inuktitut conversation, a team member described what was happening as “talking about how they’re related, and not strangers with each other.” These relational ties were expressed by one woman’s escort as likely to shape the boarding home experience that ensues, as “now that they’ve met each other, they won’t feel so much as strangers to each other in the boarding home . . . It’s a lot better.” Sewing together was observed to be generative of connections to carry forward beyond the session. Sewing as a strategy for arts-based inquiry, then, involved women gathering and having time and space to relate. After meeting and sewing together, even local team members discussed making *kamiks* (boots) together outside of the sewing sessions.

#### Sewing as “keeping our tradition going.”

“Keeping our tradition going” or a living of Inuit tradition—these were ways women described what sewing was to them. In the context of sewing together, women shared the rich traditions Inuit have around sewing, and the depth and breadth of knowledge Elders have about sewing. It was discussed as a highly developed art, associated with much skill and technicality, and for which there exist knowledge holders in Inuit communities. Women discussed differences in sewing techniques from one community to another, as well as across regions and territories in Inuit Nunangat. Just as Qikiqtaaluk Region communities are diverse, so also are the expressions of sewing, according to participants. The patterns created, used, and passed among Inuit and generationally, as well as the styles and types of sewing projects, were discussed as reflecting a particularity to place and people. In the context of the sewing groups, women also shared patterns and practices, asked sewing questions, and taught one another. Sewing, then, was described and enacted as a way to teach and carry on Inuit culture, and embody Inuit knowledge and traditions. Women expressed enthusiasm for this collective learning, for—as one participant shared—“I’m glad I came. It’s gonna motivate me to make an amauti!”

### Pragmatic Lessons Learned on Process and Power

**Figure 5.**
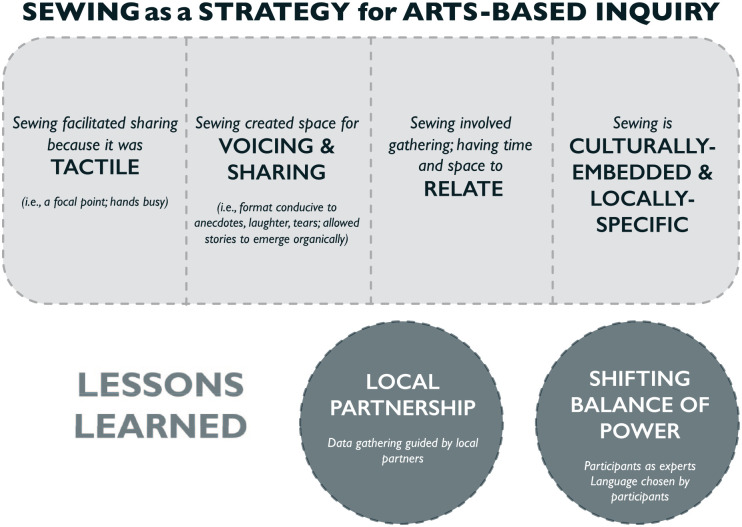
Visual synthesis of results, including what sewing facilitated in the data gathering process and pragmatic lessons learned from this research. *Note.* Both sections of results demonstrate how sewing facilitated data gathering among Qikiqtaaluk Inuit in the present study and also the possibilities for arts-based inquiry when utilized in other research contexts.

In addition to the significance of sewing to Inuit women, and what sewing together facilitated in the research process, this study also revealed some “lessons learned” (Figure 5). These observations on the process, including both challenges and strengths associated with this sewing method, emerged as the research team reflected on the sessions.

#### Partnership as critical to conversation

At times, conversation appeared stilted by what a Settler team member perceived as a benign question from her cultural lens. Conversely, other questions she considered surface-level elicited unexpectedly rich responses. Upon reflection, we noted the critical place of partnership in this research—and, perhaps, more broadly in research that crosses cultural, socioeconomic, and Indigenous-Settler bounds (Castleden et al., 2008; Fisher & Ball, 2003). Indeed, this research would not have existed in the form it did as an isolationist research endeavor. As an Inuit and Settler team, we worked in partnership from the research design to the iterative refinement of question topics, and to data gathering, interpretation, and analysis. The nuances of how and when to ask questions, and what questions were appropriate, were also engaged and directed collaboratively. Furthermore, an Inuk team member consistently adjusted and fine-tuned the phrasing of questions to invite conversation in ways that resonated with Inuit culture and respected people’s dignity and differing comfort levels in sharing.

#### Language and power in the sewing process

Throughout the sessions, a Settler team member questioned how power existed, the effects it had on the process, and how she might creatively engage the power imbued in her social identity and redress power imbalances (Castleden et al., 2008; Wallerstein & Duran, 2006). Importantly, participants could choose what language to speak, thereby controlling the course of conversation, and helping to shift the balance of power to them (Wallerstein & Duran, 2006). Participants had further agency in the discussion, then, given that language can affect presentation of self, as well as one’s processing of—and contributions to—conversation (Du, 2015). This point of power had particular relevance when considering the extent of difference between English and Inuktitut languages, and the complexity of Inuktitut, whereby concepts are likely to be described and understood very differently than in English (Joanis et al., 2020); women were freer, then, to contribute to conversation using the language they felt would best convey what they wished to share.

Beyond language, a team member’s positionality as a White Settler academic researcher also created multiple, interrelated, and reinforcing gradients along which power affected interactions with participants. She was also not skilled in the act of sewing nor a mother—two facets for which she was evidently not “expert” and instead positioned as learner (Sword, 1999). Participants knew this, as she shared her lack of sewing and birthing knowledge, stumbled through sewing with skins, and often needed to ask for help. More than once, women articulated insights they wanted to pass to the “non-mother” in the room, in the form of cautionary tales or advice for healthy pregnancy, should she be pregnant in the future. The session format was conducive to this “researcher as learner” posture, possibly more than other methods.

## Discussion: Sewing as Facilitative of Data Gathering

*Some of these women haven’t sewn before. I want our tradition alive as long as possible. ’Cause our ancestors survived without all the equipment we have today. They survived on just the land. They carved needles, they made ulus. I feel spoiled today because we have sewing machines, we have needles, we have all these things to make it easier for us. Our ancestors would have been proud, if they’d see what I can make today and provide for everyone. And that’s a big part of who we are as Inuit. It identifies how powerful our ancestors were. And not only that, whatever we create, it becomes beautiful! That’s what encourages me in passing it on, ’cause if you practice, you’ll only get better each time you try. I want these women to represent our culture, and only get better through practice*. (Naomi Tatty, Inuk team member and co-author, on sewing and Inuit tradition)

As illustrated by these results, sewing as a strategy for arts-based inquiry has broader applicability to other contexts, due to its flexible and tactile nature, and in how it creates space for voicing and sharing, as well as connection among participants. Importantly, these results can be situated among literature on intersecting and interrelated “projects” that Indigenous communities are engaged in, outlined by Linda Tuhiwai Smith (2012), as well as prominent literature on Indigenous knowledge systems (Simpson, 2014), research methods (Wilson, 2008), and methodologies (Aariak, 2018; Chilisa, 2011; Kovach, 2009). This sewing strategy—with Qikiqtaaluk Inuit in the present study—is, thus, one example of how arts-based approaches embedded in place- and culturally specific forms of knowledge sharing have potential to contribute to decolonizing health research.

The use of sewing as a strategy for arts-based inquiry was engaging for women. The research method itself—not just any future outcome or output—was identified as meaningful for participants, as it resonated with cultural values and locally specific forms of communicating and knowledge-sharing (Emanuelsen et al., 2020). In *The Hands’ Measure*, Eva Aariak (2018) says of sewing: “Taught in its proper way, the wide range of Inuit sewing practices incorporates trusted, age-old knowledge of the environment, of the seasons, and of the life cycles and anatomy of the animals on which we depend” (p. 13). The specific materials and other sewing resources used depended on what is—and has been historically—available in one’s specific environment, and clothing is known to vary by season, according to the land-based activities it would be needed for (Bennett & Rowley, 2004, p. 322). Inuit sewing practice, then, reflects a connectedness to community, lands, and place, and relates to the decolonizing “project” of connecting (Smith, 2012).

Moreover, Karetak et al. (2017) state that, for Inuit, “knowledge without application has no value” (p. 19). Rather, knowledge is produced and sustained through embodied practices such as sewing; it is experiential and “lived-in” (Simpson, 2014)—a means of “wearing your teachings” (Simpson, 2014, p. 11, quoting Elder Edna Manitowabi). Leanne Simpson (2014) describes this “coming to know” from an Indigenous perspective as “tak[ing] place in the context of family, community and relations” and a process that is “learner-led . . . the pursuit of whole body intelligence” (p. 7). This “doing” reflects an Inuit worldview of how knowledge and wisdom is formed, from the tactile and embodied practices of life (Karetak et al., 2017; Kovach, 2009; Ritenburg et al., 2014). As illustrated in the results, the experience of sewing was not a transcending of reality; rather, reality was described as being grounded by this tactile practice (Healey, 2019). The present was portrayed as focused and lived more vibrantly. The physicality between the sewing materials, the participant, and the shared sewing experience may have facilitated an embodiment of childbirth narratives and perspectives, as the tactile nature of the craft was familiar, focusing, and grounding (Bunce et al., 2016; Emanuelsen et al., 2020): Eva Aariak (2018) describes “efforts to become proficient in skin sewing” as “ma[king] us feel centred and more grounded as Inuit” (p. 13). Importantly, as reflected in the IQ (Inuit knowledge and worldview) principle of *Pilimmaksarniq*, Inuit value practicing and honing skills, such as sewing, which contribute to family and community life and wellness (Karetak et al., 2017). Donald Uluadluak (2017) references materiality when describing this learning-by-doing principle: “What we call *pilimmaksarniq* is when you are training anyone using concrete materials and tools that children can feel and practise with” (p. 165). Thus, sewing as a strategy for arts-based inquiry aligns with an Inuit epistemological, as well as ontological, framework of coming to know and be through making and doing (Healey, 2019).

Sewing as a strategy for arts-based inquiry can be further situated within Healey and Tagak’s (2014) framework for health research methodology in an Inuit context, by creating a relational research space and co-creating research through storytelling and sharing of knowledge. This “method of discovering relations,” evident in the sewing sessions, reflects the centrality of relationships to the lives of many Indigenous Peoples (Wilson, 2008, p. 84), including Inuit. Relational, storytelling methodologies, then, such as sharing circles (Waddell et al., 2020) or the use of yarning in interviews (Byrne et al., 2021), are often used in Indigenous research contexts. The voicing of stories, described in the results, can also be situated within the decolonizing “project of storytelling,” wherein Smith (2012) states that “intrinsic in story telling is a focus on dialogue and conversations amongst ourselves as indigenous peoples, to ourselves and for ourselves” (p. 146). Among Inuit, this sharing of stories while sewing reflects the Inuit concept of *Unikkaaqatiginniq*, related to storytelling, which is central to Inuit ways of knowing and being (Healey & Tagak, 2014). Embedded in this philosophy is the understanding that story is a powerful mode for knowledge-generation and knowledge-sharing (Healey & Tagak, 2014). Storytelling often emerges from tactile “doing”—such as sewing, carving, or other forms of crafting—among Inuit (Healey, 2019; McGrath, 2011). Consequently, arts-based inquiry, including sewing, may be an effective modality for facilitating and supporting data gathering both with Inuit and in other research contexts, particularly when processes of making and doing align with modes of conversation, knowledge-creation, and knowledge-sharing in the cultural context of the research.

Importantly, the use of arts-based approaches as pathways to knowledge-generation and knowledge-sharing is not new in Nunavut. For instance, Qaujigiartiit Health Research Centre in Iqaluit has long used arts-based methods to facilitate community-based health research (G. Healey Akearok, personal communication, December 16, 2019). Likewise, community sewing programs, such as that of the Rankin Inlet Friendship Centre (Greer, 2019) and through the Tukisigiarvik Centre in Iqaluit (City of Iqaluit, 2012), are spaces where Inuit knowledge of sewing is mobilized and practiced, and the Mittimatalik Arnait Miqsuqtuit Collective in Pond Inlet continues to explore the rich possibilities for knowledge-sharing that exist at the interface of an emerging digital media landscape in Nunavut and skills like sewing (Wachowich, 2018). Accordingly, while application of the arts for knowledge-creation and knowledge-sharing is common and important practice in Nunavut, arts-based approaches are less prevalent in the peer-reviewed methodological literature in this region, which this article aims to contribute to.

Our case study of sewing also fits within a broad spectrum of arts-based health research approaches that are increasingly, and regularly, utilized in other research contexts. For instance, body mapping and drama are used as an arts-based sexual health intervention and method to facilitate data collection in the Northwest Territories (Lys et al., 2018). Related research on illness prevention (Helm et al., 2015) with Indigenous youth in a global context further underscores how arts-based methods have been used effectively as engaging and empowering tools for public health research, particularly with Indigenous populations (Fitzpatrick & Reilly, 2019; Hammond et al., 2018). Also of significance to public health, photovoice is an arts-based method that has been used to evaluate access to and use of community programs (Ford et al., 2013; Lardeau et al., 2011), and more broadly to explore concepts of health among Indigenous children and youth (Kellock, 2011). Within this array of research contexts, arts-based methods, and health-related topics, our case study of sewing contributes an example of the arts as facilitative of research with Indigenous women on lived experiences of health care and perspectives on health and wellness (Poudrier & Mac-Lean, 2009).

Thus, sewing is but one example of a strategy for arts-based inquiry that may contribute to a decolonizing research agenda beyond an Inuit context. As there was no structured interview guide used in the sewing sessions, women were freer to share on their own terms, when and if they felt comfortable doing so. Overall, the design was flexible and adaptable; it was iteratively created as the research team debriefed what went well and implemented changes for subsequent sessions to facilitate further opportunities for women to share in ways that resonated with them. Labonté (2011), quoting Paulo Freire, states that “the first act of power people can take in managing their own lives is ‘speaking the world,’ naming their experiences in their own words under conditions where their stories are listened to and respected by others” (p. 156). Indeed, this strategy attempted to shift the power imbued in a researcher’s identity to participants, by creating a context where they could “speak their worlds,” less driven by the parameters of a researcher’s questioning. It involved shared experience of an activity together, whereby participants’ stories and perspectives could emerge over the course of time and companionable silence. The design was also driven by an Inuk team member with sewing expertise, who provided leadership to the sewing process and asked the majority of questions. This attempt to redress power imbalances within the research process, challenging the distinctions between “researcher” and “researched” (Smith, 2012), and prioritizing Inuit forms of knowledge-creation and sharing, may, in part, illustrate how strategies for arts-based inquiry, like sewing, have potential to contribute to a decolonizing research agenda.

## Sewing as a Strategy for Arts-Based Inquiry: Implications for Health Research and Practice

This study illustrates that, in light of calls for Inuit-led and Inuit-directed research (Inuit Tapiriit Kanatami, 2018), strategies for arts-based inquiry—such as sewing—offer possibilities for expanding Inuit leadership in research design and implementation, and rooting research in an Inuit epistemological and ontological framework. Hammond et al. (2018) situate arts-based inquiry in relation to many Indigenous ontologies, which often involve this co-creation of knowledge from experience—from *doing*. Thus, our case study of sewing with Inuit women in Iqaluit, Nunavut, more broadly illustrates the potential of arts-based inquiry to resonate with local ways of knowing and being in the context of health research with other Indigenous populations, thereby situating research to respond more effectively to a decolonizing research agenda.

## Notes

1. Obstetric evacuation refers to a model of centralized maternity care in which pregnant women travel by plane from their home communities elsewhere, typically to deliver their babies in a more centrally located health care facility.
